# Squamous cell carcinoma during long term hydroxyurea treatment: A case report

**DOI:** 10.1016/j.ijscr.2021.106160

**Published:** 2021-06-30

**Authors:** Ouassime Kerdoud, Rachid Aloua, Amine Kaouani, Ousmane Belem, Faiçal Slimani

**Affiliations:** a Department of Stomatology, Oral and Maxillofacial Surgery, Hospital of August 20th, University Hospital, Casablanca, Morocco; b Faculty of Medicine and Pharmacy, Hassan 2 University of Casablanca, Casablanca, Morocco; c Oral and maxillofacial surgery department, CHU Yalgado, Ouedraogo, Ouagadougou, Burkina Faso

**Keywords:** Hydroxyurea-induced, Skin cancer, Horn, SCC

## Abstract

**Background:**

Hydroxyurea (HU) is a ribonucleotide diphosphate reductase inhibitor that interferes with the S phase of cell replication and inhibits DNA synthesis, with limited or no effect on RNA or protein synthesis. The cutaneous side effects of hydroxyurea treatment are diverse and frequent. Squamous cell carcinoma is one of the most challenging side-effect.

**Case presentation:**

The authors report the case of a healthy 59-year-old woman on long-term therapy with Hydroxyurea 500 mg daily for essential thrombocytosis, presented with a painless slow-growing lesion of the jaw that had persisted and increased in size for six months, the appearance of the lesion is correlated to the administration of the hydroxyurea treatment. Clinical examination revealed a large nodular lesion 4 × 4 cm with irregular borders of the right cheek, infiltrated into underlying tissue, the lesion extending to the free border of the right lower eyelid without sensory disturbances or diplopia. The surgery was indicated. The surgical procedure had the aim of the restoration of the anatomic landmarks after a large excision of the tumor and reconstruction of full-thickness eyelid defect with a local flap under general anesthesia.

**Conclusion:**

Maxillofacial surgeons must be aware of the side effects of hydroxyurea including facial cancer, ulceration, etc. Rigorous follow-up of patients on hydroxyurea is required.

## Highlights

• The cutaneous cancers associated with the use of hydroxyurea are often multiple.
• They typically appear on sun-exposed surfaces and may occur in patients who have other cutaneous manifestations of hydroxyurea therapy.
• Follow-up is necessary to reveal recurrence.

## 1 Introduction

Hydroxyurea (HU) is a ribonucleotide diphosphate reductase inhibitor that interferes with the S phase of cell replication and inhibits DNA synthesis, with limited or no effect on RNA or protein synthesis [1], [2], [3]. It is one of the mainstays of the therapeutic armamentarium for the treatment of myeloproliferative disorders (MD) and the first-line treatment for intermediate to high-risk essential thrombocythemia/ sickle cell disease [4] Although HU is easy to use and effective and has high tolerance, there have been numerous reports of cutaneous complications during long-term therapy with HU. The cutaneous side effects of hydroxyurea treatment are diverse and frequent. Squamous cell carcinoma is one of the most dreaded effects [5], [6], [7], [8]. The authors report the case of a patient with skin carcinomas after treatment with hydroxyurea.

## 2 Case report

Our work is a single case report and has been reported in line with the SCARE criteria [9].

**Fig. 1:**
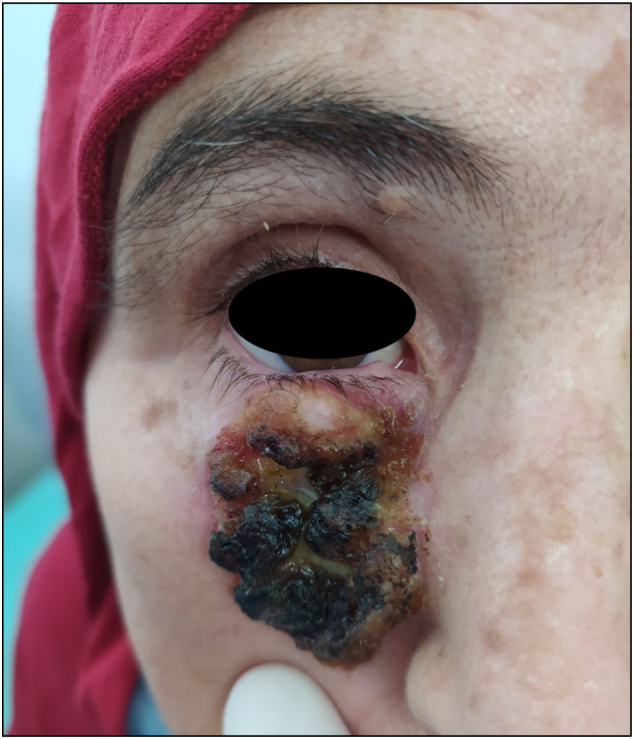
SCC of the right cheek (T3, N0, M0).

**Fig. 2:**
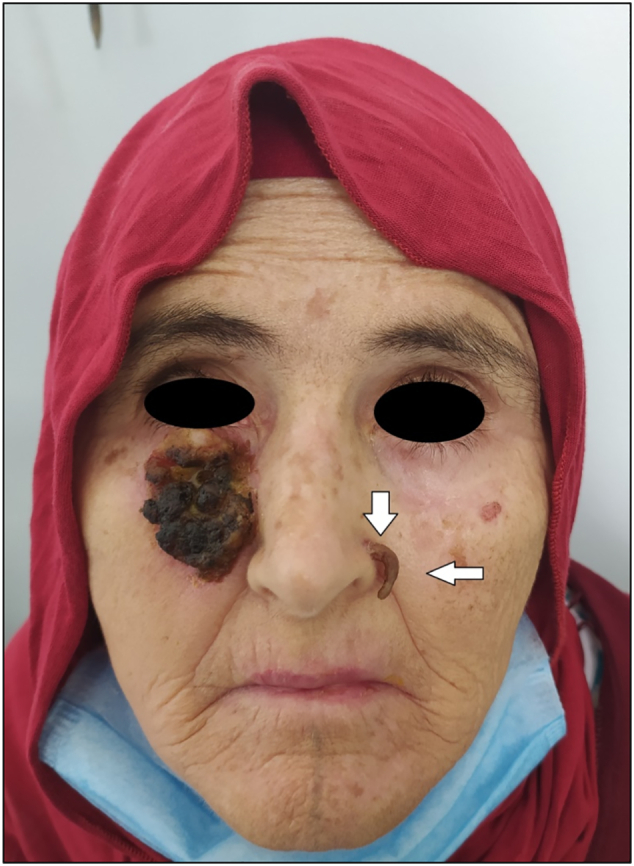
The black horn on the left side of the nose (actinic keratosis).

A healthy 59-year-old woman on long-term therapy with Hydroxyurea 500 mg daily for essential thrombocytosis, presented with a painless slow-growing lesion of the jaw that had persisted and increased in size for six months, the appearance of the lesion is correlated to the administration of the hydroxyurea treatment. There was no history of local trauma, radiotherapy, HPV infections, or family history of malignancy. She was referred to our department's consultation for specialized care. No other personal or family history was raised during the patient interrogation. Clinical examination revealed a large nodular lesion with irregular borders of the right cheek, infiltrated into underlying tissue, the lesion extending to the free border of the right lower eyelid with dimensions 4 × 4 cm, without sensory disturbances or diplopia (Fig. 1.). In the nose, black horn-shaped hyperkeratosis lesion in the nasal pyramid (Fig. 2.).

There were no palpable neck lymph nodes on the left side.

A biopsy of surgical material was performed, showing a well-differentiated squamous cell carcinoma.

The definitive diagnosis of malignancy was made; metastatic investigations were performed, including chest radiography, abdominal echography, PET scan. The results of these exams were normal.

The computed tomography (CT) was performed and found free lymph node.

On the biological level, the patient presented anemia at 7.4 g / dl for which she was transfused; a thrombocytosis at 548.000, the rest of the assessment was without particularities.

Based on the positive medical history and clinical examination, the patient was diagnosed clinically with hydroxyurea-induced squamous cell carcinoma, the hydroxyurea was ceased and the hematological therapy was switched to busulfan. The surgery was indicated and performed by the chief professor of our department who has 15 years of operative experience. The surgical procedure had the aim of the restoration of the anatomic landmarks after a large excision of the tumor and reconstruction of full-thickness eyelid defect with a local flap under general anesthesia.

Duration of surgery: 60 min; estimation of blood loss: 150 ml; duration of hospital stay: 3 days.

The patient received amoxicillin/clavulanic acid 1 g twice daily and antalgics for 8 days.

Post-operative histopathology confirmed SCC proliferation; the different margins of excision were healthy.

The multidisciplinary team deciding on the treatment options included surgeons, oncologists, radiotherapists, ophthalmologist surgeons, radiologists. The decision was made to treat the patient with the surgical approach (surgical excision of the tumor) with 1.5 cm free margins and reconstruction; the authors used the superior pedicle nasolabial island flap; with postoperative radiotherapy (PORT) and postoperative chemotherapy (POCRT). Considering the patient's age, general history, the size of the tumor, the prognosis was average.

Postoperative periods were favorable; the scar was clean and non-inflammatory.

Routine follows up 3, 6, and 12 months showed no signs of recurrence.

## 3 Discussion

The association of HU treatment with secondary skin tumors in sun-exposed areas has been widely documented in the literature [10], effectively, oral SCC secondary to HU develops after more prolonged regimens and a higher cumulative dose of HU compared with other skin tumors [11].

The association of multiple skin cancers with long-term hydroxyurea use is now recognized. Many studies have noted the late onset of subsequent skin cancers despite discontinuation of treatment [5].

It's supposed that, while tacking hydroxyurea, UV radiation provides an ideal environment for the formation of cSCC. In addition, HU can markedly elevate p53 levels in basal layer keratinocytes, which increases the risk of skin cancers; it also inhibits DNA synthesis and DNA repair in ultraviolet-irradiated human cells, interfering with cell replication in the basal layer of the epidermis [12], [13], [14], [15], [16]. This pathogenic mechanism could explain the appearance of the lesion on the face which is a photo exposed skin [17].

Actinic keratosis seems to be correlated with the administration of hydroxyurea, in this case, the association with SCC was mentioned to enhance its precancerous character [18].

The significant size of the lesion; is because the patient did not present immediately after noticing the tumor, but at a much later time [19].

A regular dermatological examination and UV protection are imperative and essential for patients on long–term hydroxyurea treatment [20], [21].

This case presentation underlines the importance and regular follow-up of these patients in the short, medium, and long term of patients receiving hydroxyurea to detect any suspicious lesion.

## 4 Conclusion

A causal relationship between long-term HU treatment and facial cancer is present and has been treated in the literature, but advanced correlation studies on larger case series are lacking. Maxillofacial surgeons must be aware of the side effects of hydroxyurea including facial cancer, ulceration, etc. Rigorous follow-up of patients on hydroxyurea is required.

## Provenance and peer review

Not commissioned, externally peer-reviewed.

## Sources of funding

The authors declared that this study has received no financial support.

## Ethical approval

Written informed consent was obtained from the patient for publication of this case report and accompanying images. A copy of the written consent is available for review by the Editor-in-Chief of this journal on request.

## Consent

Written informed consent was obtained from the patient for publication of this case report and accompanying images. A copy of the written consent is available for review by the Editor-in-Chief of this journal on request.

## Guarantor

Ousmane Belem

## CRediT authorship contribution statement

Ousmane Belem: Corresponding author writing the paper

Ouassime Kerdoud: writing the paper

Rachid Aloua: writing the paper

Amine Kaouani: writing the paper

Tarcissus Konsem: correction of the paper

Faiçal Slimani: Correction of the paper

## Declaration of competing interest

Authors of this article have no conflict or competing interests. All of the authors approved the final version of the manuscript.
